# The Effects of Cyclophosphamide on Hippocampal Cell Proliferation and Spatial Working Memory in Rat

**DOI:** 10.1371/journal.pone.0021445

**Published:** 2011-06-22

**Authors:** Laura Lyons, Maha ELBeltagy, Geoffrey Bennett, Peter Wigmore

**Affiliations:** 1 School of Biomedical Sciences, Queen's Medical Centre, University of Nottingham, Nottingham, United Kingdom; 2 Department of Anatomy, Menoufiya University, Shebin El-Koom, Egypt; University of Memphis, United States of America

## Abstract

Cyclophosphamide (CP) is a chemotherapy used in combinations that are associated with cognitive impairment. In the present study male Lister-hooded rats (n = 12) were used to investigate the effects of chronic administration of CP (30mg/kg, 7 i.v. doses, or an equivalent volume of saline) on performance in the novel location recognition (NLR) task and on the proliferation and survival of hippocampal cells. The survival of hippocampal cells dividing at the beginning of treatment was significantly reduced by CP. However, no difference was seen between CP treated and control groups for the number of cells proliferating 7 days after the final injection and both groups performed equally well in the NLR task. These results indicate that the given dose of CP acutely reduces the survival of newly born hippocampal cells. However, it does not have a longer term effect on spatial working memory or hippocampal proliferation, suggesting that CP is less neurotoxic than other chemotherapies with which it is used in combination.

## Introduction

Patients who have received adjuvant chemotherapy as part of their treatment for cancer often report problems in cognition, encompassing memory impairment, a short concentration span and general confusion [1]. These effects can last up to several years after completion of the treatment [2], [3]. However combination therapies are often used in clinical treatment, so the actions of individual drugs are unclear.

In a recent review of rodent models of the cognitive effects of chemotherapy by Seigers and Fardell [4], the majority of investigations found that chemotherapy has a negative effect on different aspects of learning and memory. However a small number found that chemotherapy has no effect on cognition [5], [6] and one even reported an improvement [7]. Furthermore, many of these studies also found that chemotherapy reduced proliferation of neural progenitors in the dentate gyrus (DG) of the hippocampus [8], [9], [10]. This reduction in hippocampal neurogenesis has been considered as one possible cause of the cognitive impairment seen. Throughout life, neuronal progenitors in the subgranular zone of the DG divide to produce new neurones, which get integrated into existing neural circuits [11] and are thought to have a functional role in learning and memory consolidation [12], [13]. Ablation of neurogenesis by means of irradiation [14], hippocampal lesions [15], [16] or cytotoxic drugs [17], [18] has been shown to cause impairment in cognition.

Cyclophosphamide (CP), methotrexate and 5-fluorouracil (CMF) are all chemotherapy drugs, commonly used in combination to treat breast cancer [19]. This combination is reported to have an effect on cognition in human studies [20], [21], [22]. Previous work has shown that both methotrexate and 5-fluorouracil have a negative effect on memory and proliferation in the hippocampus [9], [23], [24] and the present study focuses on the effects of CP.

CP is an alkylating agent, with its metabolites causing alkyl crosslinks within and between DNA strands of dividing cells, causing them to apoptose [25]. It is able to cross the blood brain barrier [26]. In the present study CP was administered chronically in a rat model to mimic clinical administration. A dosage of 30mg/kg was chosen which is sufficient to cause weight loss but well below the predicted median lethal dose of 200mg/kg [27] and below the amount administered which causes pain or cystitis [28]. One study showed CP had an increase in antiprolific action and less toxicity when adult rats were dosed at 14.00 h compared with 8.00 h [29], so in the present study all injections were administered between 14.00 and 16.00 h.

In the present study, the novel location recognition (NLR) task [30] was used to test spatial memory 6 days after the final CP injection. Ki67 is a protein which is expressed in all stages of the cell cycle [31] and was used to quantify cells which were proliferating in the DG at the end of the experiment. To investigate the effect of CP on the survival of newly generated hippocampal cells, bromodeoxyuridine (BrdU) was injected at the beginning of CP treatment to be incorporated into cells proliferating at that time. The surviving cells which expressed BrdU at the end of the experiment were quantified.

## Materials and Methods

### Ethics statement

Principles of laboratory animal care in this study were in accordance to UK Home Office Guidance regulations, within the “moderate” severity band, with approval from the University of Nottingham ethical committee board under permit number 40/2715. Throughout the experiment, discomfort to animals was kept to an absolute minimum. Animals remained in good health throughout the study and never dropped more than 10% of their highest body weight.

### Animals and treatment

Male Lister-hooded rats (125–150g; Charles River, UK) were administered CP (30mg/kg, 7 i.v. doses, each 2 days apart, into the tail vain , at a volume of 1.5ml/kg, dissolved in 0.9% sterile saline; Sigma Aldrich, UK) or equivalent volume of 0.9% sterile saline (both groups n = 12). Immediately after the first injection, BrdU was administered to both groups (250mg/kg, i.p., at a volume of 5ml/kg; Sigma Aldrich, UK). All injections were given under isofluorane anaesthesia.

Rats were housed in cages of four and maintained with a 12 h light/dark cycle (7.00/19.00 h) and food and water was provided *ad libitum.* They were weighed daily from arrival and allowed to habituate for 2 weeks prior to drug administration.

### NLR spatial working memory task

The NLR test was adapted from Dix and Aggleton [30] and commenced 5 days after the final CP injection as described by Lyons et al. [8]. In brief, rats were habituated to an arena for 30 min, 24 h prior to testing and for a further 3 min, 5 min prior to familiarization trial. In the 3 min familiarisation trial, rats were placed in the arena to explore two identical objects in different locations. Rats were removed for a 15 min retention period and then reintroduced to the arena for the 3 min choice trial in which one object had been moved to a different location. Exploration time of both objects in both trials was recorded blind twice and averaged using a stopwatch from digitised recordings.

### Brain tissue preparation and immunohistochemistry

The day after behavioural testing was completed, rats were put down by rapid stunning and cervical dislocation. Tissue preparation and staining for BrdU and Ki67 was performed as described in Lyons et al. [8]. Briefly, brains were removed and cryopreserved in 30% sucrose solution at 4°C then snap frozen. Microtome sections (20 µm) were stored at −20°C until used for immunohistochemistry.

Every 20^th^ section throughout the entire length of the dentate gyrus was selected [32]. Briefly, sections were incubated with polyclonal sheep BrdU primary antibody (1∶100; Abcam, UK) overnight followed by Alexa 488 donkey anti-sheep secondary antibody (1∶300; Invitrogen, UK) or with monoclonal mouse Ki67 primary antibody (1∶100; Vector laboratories, UK) for 1 h, followed by 1 h incubation with Alexa 555 donkey anti-mouse (1∶300; Invitrogen, UK). Sections were mounted with media containing (diamidinophenylindole) DAPI (1.5 µg/ml) nuclear marker (Vector laboratories, UK).

BrdU and Ki67 positive cells which co-localised with the DAPI nuclear staining within the SGZ of both hippocampal blades were counted on a fluorescence microscope at 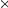
40 magnification. By combining cell counts per section for the whole dentate gyrus and multiplying by 20, the total number of co-stained cells was estimated [33]. All counting was performed blind.

### Statistical analysis

Statistical analysis and graphs were created using GraphPad Prism 5 and significance was regarded as *p*<0.05. Body weight was analysed using two-way repeated measured ANOVA. Student's paired *t* tests were used to compare exploration times of animals in the familiarisation and choice trials. Preference indices (PI) were created by expressing time spent exploring the object in the novel location as a percentage of the sum of exploration time of novel and familiar locations in the choice trial, to create a single value to compare between groups [17]. PI was compared to 50% chance using a one-sample *t* test. Student's unpaired *t* tests were used to compare PI, total exploration time and cells counts between the groups.

## Results

**Figure 1:**
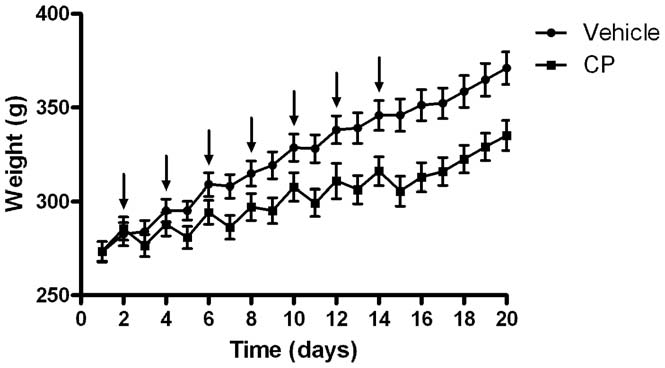
Body weights. Body weights of rats (mean ± SEM) throughout the study. Arrows indicate CP (30mg/kg)/saline injections.

Both treatment and time had a significant effect on body weight (*F*_1,418_ = 5.51, *p*<0.05 *F*_19,418_ = 367.9, *p*<0.0001 respectively, two-way repeated measures ANOVA, Figure 1) and a significant effect of treatment × time interaction was also confirmed (*F*_19,418_ = 30.23, *p*<0.0001). Animals remained in good health throughout the study and never dropped more than 10% of their highest body weight.

**Figure 2:**
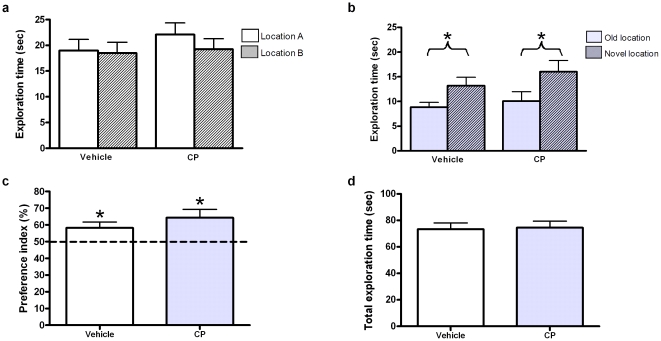
Novel location recognition (NLR) task. Mean exploration times (mean ± SEM) of the rats for each object in the familiarisation (a) and choice (b) trials of the NLR task. There was no significant difference in exploration time of either object for both groups in the familiarisation trial (*p*>0.05). In the choice trial, both groups spent significantly longer exploring the object in the novel location (*p*<0.05). Preference indices (PI, (c), mean ± SEM) were created by expressing time spent exploring the object in the novel location as a percentage of the sum of exploration time of novel and familiar locations in the choice trial (Bruel-Jungerman et al. 2005). Both groups were significantly different from chance (*p*<0.05). The total exploration time (mean ± SEM) for both trial combined (d) did not differ significantly between groups (*p*>0.05).

The NLR task was used to test cognition and Student's paired *t* tests were used to compare exploration times of the objects. In the familiarisation trial, neither the vehicle nor CP treated group explored the objects in locations A or B for significantly different lengths of time, showing no preference for either location (*p*>0.05, Figure 2a). In the choice trial, both groups preferentially explored the object in the novel location (*p*<0.05, Figure 2b), indicating no memory impairment. The raw exploration time data was converted into PI for further analysis (Figure 2c). A Student's unpaired *t* test revealed no significant difference between the PI of the vehicle and CP treated groups (*p*>0.05) and PI of both groups significantly differed from 50% chance (both *p*<0.05), indicating neither group had impaired cognition. No difference was found in total exploration time of the vehicle and CP treated groups (*p*>0.05 Figure 2d).

**Figure 3:**
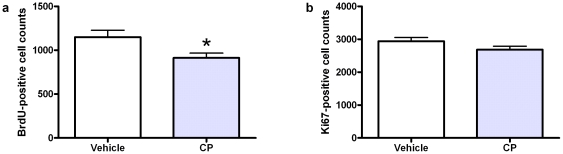
BrdU and Ki67-positive cell counts. Total number of BrdU -positive (a) and Ki67-positive (b) cells in the dentate gyrus (mean ± SEM) estimated from cell counts. Rats receiving CP had significantly fewer BrdU-positive cells (*p*<0.05) than the saline-treated control group. No significant difference was found between groups for the total numbers of Ki67-positive cells.

BrdU was injected on the day of the first CP/saline injection to investigate the survival of the cells which were dividing at that time. Animals receiving CP had significantly fewer BrdU-positive cells in the DG (*p*<0.05, Student's unpaired *t* test, Figure 3a). The most likely explanation for this is that CP treatment reduced the survival of the cells which were dividing at the start of treatment. Alternatively, it is possible that CP might have transiently increased the rate of proliferation of these hippocampal cells, causing the BrdU to become diluted and undetectable. No significant difference was found between groups for the amount of Ki67-positive cells in the DG (*p*>0.05, unpaired Student's *t* test, Figure 3b), suggesting that CP does not affect cells dividing in the DG 7 days after the final injection.

## Discussion

In the present study a rat model was used to investigate the effects of CP on cognition and the survival and proliferation of newly generated hippocampal cells.

The NLR task was chosen to test spatial working memory, as it is hippocampal dependent [34] and it does not rely on any positive or negative reinforcers which may confound results. The results showed that animals who had received a series of CP injections were still able to distinguish an object in a novel location from that in a familiar, so did not differ from the control group. This indicates that the dose and administration of CP used in the present study did not affect the rats' ability in this task. Other studies using rodent models within the literature have shown a mixture of results in regard to the effect of CP on cognition. Lee at al. [7] found that 4, 100mg/kg doses of CP, 4 weeks apart, caused an improvement in Morris water maze performance in rat. However, other authors giving 4 weekly doses of 25mg/kg CP found an impairment in a passive avoidance test a week after treatment in female rats [35]. Furthermore, Macleod et al. [6] found a sub-chronic weekly dose of 40mg/kg of CP impaired context specific, but not cue specific conditioned emotional response in rat a week after the final injection. These differences may be explained by the different dosages and different behavioural tests used. It would be interesting to use further behavioural tests to investigate the effect of dosing regimen used in the present study on different cognitive domains in rat. Studies on mice have found an acute affect (within 24 hours) on memory but recovery within a period of days [36], [37]. The results in the present study demonstrate that CP causes no deficit in the NLR task 6 days after the final CP injection. However this does not preclude the possibility that CP may have acute effects on memory which recover over a short time period. It is also possible that CP may have longer term effects which have not been examined in the present study.

New neurons in the DG have been shown to be preferentially used in spatial learning tasks [38] and reductions in DG neurogenesis impair the ability of animals to perform these tasks [39]. It is estimated that over 80% of dividing cells in the subgranular are destined to become dentate gyrus neurones [40]. In the present study, the number of cells proliferating (Ki67-positive) in the subgranular zone of the DG, a week after the final injection, was not affected by CP compared to the control group, although the number of BrdU-positive cells was significantly reduced. This reduction suggests that the survival of the cells which were dividing at the beginning of the experiment was lower in rats that received CP, indicating the drug is cytotoxic to newly generated hippocampal cells. This might be an explanation for the acute effects on behaviour found in some studies [36], [37]. This conclusion is in line with recent studies showing that cell proliferation is reduced the day after CP administration [26] but gradually recover over the following days [36], correlating with cognitive performance.

It would be interesting to look at the effects of CP on cognition, cell proliferation and survival over a longer time period as another alkylating agent, thioTEPA, caused an initial reduction in hippocampal cell proliferation in mice, followed by a transient 3 week recovery. This in turn was followed by a long term deficiency in cell proliferation lasting for 3 months and these deficiencies were roughly correlated with spatial cognitive decline [41].

Collectively, the results of different studies of CP on cognition and neurogenesis, still do not paint a clear picture. Drug delivery, with respect to route of administration, dosage and time course has differed between studies as have the behavioural tests used. Evidence from the previous studies suggests that CP may have an acute effect on cells proliferating in the subgranular zone of the adult DG during which time animals may display cognitive deficits. However, it appears likely that the reduction in cell proliferation and spatial cognition is subtle and reversible. CP is broken down by aldehyde dehydrogenase 3 present in the brain which converts CP into non-toxic metabolites [42]. The presence of this enzyme may make the effects of CP relatively short lasting compared with other chemotherapy agents.

CP is often administered with methotrexate and 5-fluorouracil, a combination known as CMF. This drug cocktail has been associated with cognitive impairment in patient studies [20], [21] and several rodent models have been used to investigate the individual drugs. In the majority of studies, 5-fluorouracil has been shown to chronically impair memory and reduce proliferation in the DG of the hippocampus for weeks after treatment has ended [9], [43], [44]. Likewise, the negative effect of methotrexate on cognition and proliferation has also been shown to last for weeks [5], [8], [24], [44]. Indeed, previous experiments within our laboratory have shown that both 5-fluorouracil and methotrexate cause rats to be impaired in the NLR task used in the present study and reduce both the proliferation and survival of hippocampal cells [8], [9]. If these results are translatable to humans, they suggest that although CP may acutely impair spatial cognition and reduce the survival of newly generated hippocampal cells, this is reversible in a matter of several days and it is likely to be other chemotherapy drugs causing the longer-term impairment.
